# Transcatheter aortic valve replacement in obese patients: procedural vascular complications with the trans-femoral and trans-carotid access routes

**DOI:** 10.1093/icvts/ivab354

**Published:** 2021-12-23

**Authors:** Alberto Alperi, Angela McInerney, Thomas Modine, Chekrallah Chamandi, Jose D Tafur-Soto, Marco Barbanti, Diego Lopez, Francisco Campelo-Parada, Asim N Cheema, Stefan Toggweiler, Francesco Saia, Ignacio Amat-Santos, Juan F Oteo, Viçent Serra, Maciej Dabrowski, Ramzi Abi-Akar, Natalia Giraldo Echavarria, Roberto Valvo, Javier Lopez-Pais, Anthony Matta, Mobeena Arif, Federico Moccetti, Miriam Compagnone, Siamak Mohammadi, Luis Nombela-Franco, Josep Rodés-Cabau

**Affiliations:** 1 Quebec Heart & Lung Institute, Laval University, Quebec City, Canada; 2 Cardiovascular Institute, Hospital Clinico San Carlos, Madrid, Spain; 3 Centre Hospitalier Universitaire de Lille, Lille, France; 4 Hôpital européen Georges-Pompidou, Paris, France; 5 The Ochsner Clinical School, Ochsner Medical Center, New Orleans, LA, USA; 6 Ferrarotto Hospital, University of Catania, Catania, Italy; 7 CIVERCV, Instituto de investigación de Santiago (IDIS), Hospital Clínico Universitario de Santiago de Compostela, Santiago de Compostela, Spain; 8 Cardiology Department, Rangueil University Hospital, Toulouse, France; 9 Division of Cardiology, St Michaels Hospital, Toronto, Canada; 10 Heart Center Luzerne, Luzerner Kantonsspital, Lucerne, Switzerland; 11 Cardiology Unit, Cardio-Thoracic-Vascular Department, University Hospital of Bologna, Policlinico S. Orsola—Malpighi, Bologna, Italy; 12 CIBERCV, Instituto de Ciencias del Corazón (ICICOR), Hospital Clínico Universitario de Valladolid, Valladolid, Spain; 13 Department of Cardiology, Hospital Universitario Puerta de Hierro, Majadahonda, Spain; 14 Hospital General Universitari Vall d’Hebron, Barcelona, Spain; 15 Department of Interventional Cardiology and Angiology, National Institute of Cardiology, Warsaw, Poland; 16 Hospital Clínic Barcelona, Barcelona, Spain

**Keywords:** Transcatheter aortic valve replacement, Transfemoral, Transcarotid, Obesity

## Abstract

**OBJECTIVES:**

Obesity may increase the risk of vascular complications in transfemoral (TF) transcatheter aortic valve replacement (TAVR) procedures. The transcarotid (TC) approach has recently emerged as an alternative access in TAVR. We sought to compare vascular complications and early clinical outcomes in obese patients undergoing TAVR either by TF or TC vascular access.

**METHODS:**

Multicentre registry including obese patients undergoing TF- or TC-TAVR in 15 tertiary centres. All patients received newer-generation transcatheter heart valves. For patients exhibiting unfavourable ileo-femoral anatomic characteristics, the TC approach was favoured in 3 centres with experience with it. A propensity score analysis was performed for overcoming unbalanced baseline covariates. The primary end point was the occurrence of in-hospital vascular complications (Valve Academic Research Consortium-2 criteria).

**RESULTS:**

A total of 539 patients were included, 454 (84.2%) and 85 (15.8%) had a TF and TC access, respectively. In the propensity-adjusted cohort (TF: 442 patients; TC: 85 patients), both baseline and procedural valve-related characteristics were well-balanced between groups. A significant decrease in vascular complications was observed in the TC group (3.5% vs 12% in the TF group, odds ratio: 0.26, 95% CI: 0.07–0.95, *P* = 0.037). There were no statistically significant differences between groups regarding in-hospital mortality (TC: 2.8%, TF: 1.5%), stroke (TC: 1.2%, TF: 0.4%) and life-threatening/major bleeding events (TC: 2.8%, TF: 3.8%).

**CONCLUSIONS:**

In patients with obesity undergoing TAVR with newer-generation devices, the TC access was associated with a lower rate of vascular complications. Larger randomized studies are warranted to further assess the better approach for TAVR in obese patients.

**Figure:**
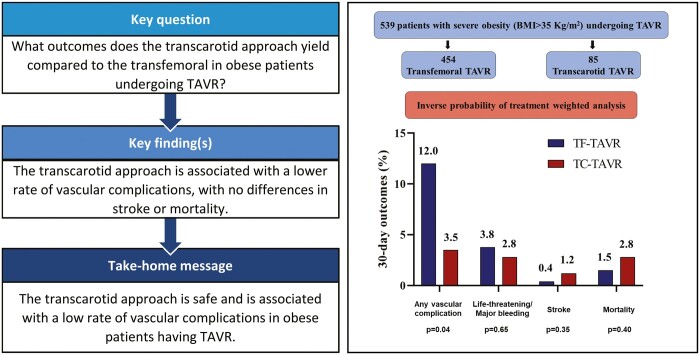

## INTRODUCTION

Transcatheter aortic valve replacement (TAVR) has been established as a first-line therapeutic option for high-risk and elderly patients with symptomatic severe aortic stenosis [1–3]. However, patients with at least grade II obesity [those exhibiting a body mass index (BMI) >35 kg/m^2^] have been underrepresented in the main TAVR versus surgical aortic valve replacement trials. The prevalence of obesity has dramatically increased in western countries over the last 30 years, and it has been estimated that >6% of adults in North America have severe obesity [4, 5]. In addition, previous observational studies showed that the prevalence of severe obesity in real-world TAVR populations was close to 15% [6, 7].

The transfemoral (TF) access is well-recognized as the default approach for TAVR procedures. However, it is widely accepted that femoral vascular access management and hemostasis are more challenging in obese patients because of their inherent anatomic features. In fact, obesity has been reported to be an independent predictor for vascular access complications in patients undergoing TF coronary interventions [8, 9]. In the TAVR field, the transcarotid (TC) and transaxillary approaches have been associated with improved clinical outcomes compared to the more invasive transapical and transaortic alternatives [10]. Moreover, it has been reported that the main safety and efficacy outcomes obtained with TF-TAVR could be mimicked by TC-TAVR in a real-world population [11, 12, 13]. Indeed, the TC approach has been associated with a very low rate of vascular and bleeding complications [12], and this may represent a major advantage in the challenging group of TAVR candidates with important obesity. Thus, we sought to compare the early clinical outcomes in obese patients (at least grade II obesity) undergoing TAVR either by TF or by TC vascular access, with special focus on vascular complications.

## MATERIALS AND METHODS

### Ethics statement

The study was performed in accordance with the institutional review board of all participating centres, which granted the approval for the study. All patients provided written informed consent for the procedures.

### Study population

This was a multicentre, observational study involving 15 tertiary-care hospitals from Europe and North America. Consecutive patients with at least grade II obesity (i.e. BMI >35 kg/m^2^) who underwent TF- or TC-TAVR between March 2015 and July 2020 were included. Patients who underwent TAVR by other approach beyond TF or TC and those in whom TF-TAVR was performed with the use of a surgical cut-down approach were not included in the study. In addition, those patients receiving early-generation transcatheter heart valves (THVs) were excluded, aiming to improve comparability between groups and to include predominantly a contemporaneous subset of patients. The following THVs were considered to be early-generation devices: the balloon-expandable SAPIEN and SAPIEN XT (Edwards Lifesciences, Irvine, CA, USA) and the self-expandable CoreValve (Medtronic Inc., Minneapolis, MN, USA). A list of inclusion/exclusion criteria is presented in Supplementary Material, Table S1.

### Vascular access selection

Multimodality vascular evaluation was performed in all cases prior to the procedure to select the optimal vascular access, including multi-slice computed tomography of the aorta, iliac, femoral and carotid arteries, as well as Doppler sonography of the carotid and supra-aortic arch vessels. Whenever feasible, TF was considered as the first-line approach. In 3 participating centres, the TC cut-down approach was the preferred access for those patients with unfavourable iliofemoral anatomy (defined as a minimal lumen diameter below the recommended one according to the size of the selected THV or as the presence of severe calcification/tortuosity), prior peripheral lower limb vascular intervention, or significant disease of the thoraco-abdominal aorta precluding a safety passage of the THV system. Two conditions should have been fulfilled before TC-TAVR: the presence of a non-diseased common carotid artery with a minimal lumen diameter above 7 mm and the absence of contralateral significant (>50%) internal or common carotid artery stenosis.

### Procedural technique

TF cases were generally performed under local anaesthesia and conscious sedation, whereas TC cases were preferentially performed under general anaesthesia. The technique for TC-TAVR has been previously described [13]. The selection of the THV and the secondary access were left to the discretion of the operators responsible for the case.

### Study end points

Baseline, periprocedural and in-hospital data were prospectively collected in a dedicated TAVR database. The primary end point was the occurrence of in-hospital periprocedural vascular complications. Secondary end points were in-hospital all-cause mortality, stroke, life-threatening/major bleeding events, need for a new permanent pacemaker implantation and new-onset atrial fibrillation. All events were defined according to the Valve Academic Research Consortium-2 criteria [14].

### Statistical analysis

Results were displayed as number (percentage) for categorical data and as mean (standard deviation) or median (interquartile range) for continuous variables. Student’s *t*-test was used to compare normally distributed continuous variables and the Mann–Whitney *U*-test was used for continuous non-normally distributed variables. The chi-square and Fisher’s exact tests were used to compare categorical variables as appropriate. The analyses were performed according to the main TAVR access (TF versus TC).

Propensity score (PS) matching with an inverse probability of treatment weight (IPW) approach was used to adjust for differences in baseline characteristics and potential confounders that may lead to biased estimates of treatment outcomes. A PS was calculated for each patient to estimate the propensity towards belonging to a specific treatment group (TC versus TF). This was done by means of a multivariate logistic regression including the following covariates: age, sex, diabetes mellitus, hypertension, chronic obstructive pulmonary disease, coronary artery disease, peripheral vascular disease, cerebrovascular disease, prior coronary artery bypass graft, prior valvular cardiac surgery, prior atrial fibrillation, New York Heart Association class at the time of procedure, prior dialysis, sheath size and Society of Thoracic Surgeons score for predicted risk of mortality. The PS calculation allowed case-weight estimation to predict the inverse probability of having a TC approach. The case weights balanced the cohorts for an IPW analysis that included all patients with available data for the variables included in the propensity model. The adequate balancing of covariate distribution between the matched groups was numerically assessed by means of standardized means differences before and after IPW-matching, and graphically assessed by means of the box and cumulative probability plots for raw and IPW-adjusted data (Supplementary Material, Figs. S1 and S2). Then, an inverse probability of treatment weighted logistic regression was performed to determine the relation between the TAVR approach and our primary and secondary end points. This logistic regression was also adjusted for the variables with standardized mean differences > or <0.1 after IPW-matching. Data analyses were performed using STATA (v14.0; StataCorp), and *P*-values <0.05 were considered significant. The data underlying this article will be shared on reasonable request to the corresponding author.

### Data availability statement

The data that support the findings of this study are available from the corresponding author and the authors from each participating center on reasonable request.

## RESULTS

A total of 539 patients were included, with a mean BMI of 39.6 (4.9) kg/m^2^. Among them, 454 (84.2%) patients underwent TF-TAVR and 85 (15.8%) TC-TAVR. A flow chart summarizing patient selection is displayed in the graphic abstract. Baseline clinical and procedural characteristics for the un-matched cohort are displayed in Table 1. In the overall population, patients in the TF group were older (*P* = 0.01), had a higher prevalence of hypertension (*P* = 0.01) and exhibited a higher baseline left ventricle ejection fraction (*P* = 0.01); whereas patients in the TC subgroup exhibited a higher prevalence of peripheral vascular disease (*P* = 0.001) and a poorer functional class at the time of the procedure (*P* = 0.03). The balloon-expandable Sapien 3 valve was the system most commonly used in both groups (45.3% and 64.7% for TF-TAVR and TC-TAVR, respectively), followed by the self-expandable Evolut R/PRO system (40.8% and 35.3% for TF-TAVR and TC-TAVR, respectively). Larger vascular introducers were used for TF-TAVR than for TC-TAVR patients [15.1(1.8) F vs 14.6(1.2) F, *P* = 0.03]. The percutaneous access site closure management in TF-TAVR patients is displayed in Table 1. Most operators used the double-ProGlide strategy (64.8%), followed by the Prostar system (31.1%).

**Table 1:** Baseline and procedural characteristics of the overall cohort

	Entire population				
	Overall (*n* = 539), *n* (%)	TF (*n* = 454), *n* (%)	TC (*n* = 85), *n* (%)	*P*-Value	Std. dif.
Age, years	76.5 (8.1)	76.9 (8.2)	74.7 (7.5)	0.02	−0.29
Gender, female	331 (61.4)	286 (63)	45 (52.9)	0.08	−0.21
BMI, kg/m^2^	39.6 (4.9)	39.4 (4.3)	40.5 (7.1)	0.05	0.29
COPD	135 (25.1)	107 (23.6)	28 (32.9)	0.07	0.20
Diabetes mellitus	274 (50.8)	235 (51.8)	49 (45.9)	0.32	−0.12
Hypertension	492 (91.3)	436 (96)	56 (65.9)	0.001	−0.82
Previous CAD	222 (41.2)	187 (41.2)	35 (41.2)	0.99	0.01
Prior CABG	68 (12.6)	53 (11.7)	15 (17.6)	0.12	0.16
Prior valve surgery	41 (7.6)	31 (6.9)	10 (11.8)	0.12	0.17
Peripheral vascular disease	62 (11.5)	42 (9.3)	20 (23.5)	0.001	0.39
Cerebrovascular disease	43 (8)	38 (8.4)	5 (5.9)	0.44	−0.10
Atrial fibrillation	175 (32.5)	150 (33)	25 (29.4)	0.51	−0.08
Creatinine, mmol/l	110 (65)	110 (68)	110 (63)	0.94	−0.03
Dialysis	11 (2)	9 (2)	2 (2.4)	0.83	0.02
Permanent pacemaker	45 (8.4)	34 (7.5)	11 (12.9)	0.10	0.20
NYHA class ≥III	362 (67.1)	296 (65.2)	66 (77.6)	0.03	0.28
STS mortality	4.8 (3.9)	4.8 (3.9)	4.8 (4.2)	0.84	0.04
EuroScore 2	4.7 (5.7)	4.8 (5.5)	4.3 (6.1)	0.45	0.09
LVEF, %	55.1 (12.4)	55.6 (12.4)	52.3 (11.8)	0.03	−0.26
AVA, cm^2^	0.74 (0.21)	0.74 (0.21)	0.70 (0.15)	0.07	−0.25
Severe MR	14 (2.8)	13 (3.1)	1 (1.2)	0.33	−0.12
	(*n* = 504)				
Valve size				0.87	
≤26 mm	232 (61.6)	279 (61.4)	53 (62.4)		
>26 mm	207 (38.4)	175 (38.6)	32 (37.7)		−0.04
Valve type				0.01	
Sapien 3	261 (48.4)	206 (45.3)	55 (64.7)		
Evolut R/PRO	215 (39.9)	185 (40.8)	30 (35.3)		
Symetis Acurate	46 (8.5)	46 (10.1)	0		
Portico	13 (2.4)	13 (2.9)	0		
Allegra	3 (0.6)	3 (0.7)	0		
Direct flow	1 (0.2)	1 (0.2)	0		
Sheath size				0.14	
14 F	371 (69.1)	308 (68.1)	63 (74.1)		
16 F	97 (18)	80 (17.7)	17 (20)		
18 F	36 (6.7)	31 (6.9)	5 (5.9)		
20 F	32 (6)	32 (7.1)	0		
22 F	1 (0.2)	1 (0.2)	0		
Mean size, F	15 (1.7)	15.1 (1.8)	14.6 (1.2)	0.03	−0.24
Vascular closure technique for TF-TAVR (*n* = 454)	NA		NA	NA	
Prostar		142 (31.1)			
2 ProGlide		294 (64.8)			
1 ProGlide		14 (3.1)			
Manta		4 (0.9)			

AVA: aortic valve area; BMI: body mass index; CABG: coronary artery bypass graft; CAD: coronary artery disease; CI: confidence interval; COPD: chronic obstructive pulmonary disease; LVEF: left ventricle ejection fraction; MR: mitral regurgitation; NA: not applicable; NYHA: New York Heart Association; OR: odds ratio; STS: society of thoracic surgeons; TAVR: transcatheter aortic valve replacement; TC: transcarotid; TF: transfemoral.

A total of 442 (97.4%) and 85 (100%) patients from the TF- and TC-TAVR groups were included in the IPW propensity-matched analysis. After IPW adjustment both groups were comparable for the baseline characteristics, the type and the size of the valve implanted and the diameter of the therapeutic sheath used during the procedure (Tables 2 and 3).

**Table 2:** Baseline characteristics in the inverse probability of treatment weight-adjusted population

	IPW-adjusted population			
	TF (*n* = 442), *n* (%)	TC (*n* = 85), *n* (%)	*P*-Value	Std. dif.
Age, years	76.3 (9.1)	75.4 (7)	0.33	0.12
Gender, female	268 (60.5)	53 (62.5)	0.72	0.04
BMI	39.9 (4.2)	40.5 (6.9)	0.27	0.05
COPD	115 (25.8)	20 (24.1)	0.74	−0.04
Diabetes mellitus	231 (52.2)	51 (60.2)	0.18	0.16
Hypertension	403 (91.2)	78 (91.5)	0.97	0.01
Previous CAD	185 (41.8)	38 (44.5)	0.64	0.05
Prior CABG	59 (13.4)	11 (12.5)	0.83	−0.05
Prior valve surgery	32 (7.3)	7 (7.8)	0.87	0.02
Peripheral vascular disease	48 (10.9)	9 (10.8)	0.98	−0.003
Cerebrovascular disease	35 (8)	6 (7.4)	0.85	−0.02
Atrial fibrillation	146 (33.1)	30 (35.8)	0.63	0.06
Creatinine, mmol/l	111.5 (72)	116 (67)	0.56	0.06
Dialysis	10 (2.2)	2 (2.4)	0.91	0.05
NYHA class ≥III	296 (67)	58 (68.4)	0.80	0.03
STS mortality	4.7 (3.7)	4.7 (3)	0.80	−0.03
EuroScore 2	4.6 (5.5)	4.3 (5.3)	0.64	−0.07
LVEF, %	55.6 (11.9)	55.6 (10.2)	0.99	0.01
AVA, cm^2^	0.74 (0.21)	0.71 (0.15)	0.21	−0.17
Severe MR	15 (3.6)	2 (3.2)	0.83	−0.04

AVA: aortic valve area; BMI: body mass index; CABG: coronary artery bypass graft; CAD: coronary artery disease; COPD: chronic obstructive pulmonary disease; inverse probability of treatment weight; LVEF: left ventricle ejection fraction; MR: mitral regurgitation; TC: transcarotid; TF: transfemoral.

**Table 3:** Procedural characteristics of the inverse probability of treatment weight-adjusted population

	IPW-adjusted population			
	TF (*n* = 442)	TC (*n* = 85)	*P*-Value	Std. dif.
Valve size			0.73	
≤26 mm	282 (63.9)	56 (66)		
>26 mm	160 (36.1)	29 (34)		−0.04
Valve type			0.62	
Sapien 3	260 (58.6)	50 (58.4)		
Evolut R/PRO	163 (36.9)	35 (41.6)		
Symetis Acurate	13 (2.9)	0		
Portico	6 (1.4)	0		
Allegra	0	0		
Direct flow	0	0		
Sheath size			0.59	
14 F	360 (81.5)	69 (81.6)		
16 F	62 (14)	11 (12.6)		
18 F	12 (2.7)	5 (5.8)		
20 F	8 (1.8)	0 (0)		
Mean size, F	14.5 (1.2)	14.5 (1.1)	0.99	0.01
Vascular closure technique for TF-TAVR (*n* = 442)		NA	NA	
Prostar	128 (29)			
2 ProGlide	300 (67.9)			
1 ProGlide	12 (2.7)			
Manta	2 (0.4)			

Categorical data are expressed as *n* (%) with the *n* rounded up to the closes whole number.

IPW: inverse probability of treatment weight; NA: not applicable; TAVR: transcatheter aortic valve replacement; TC: transcarotid; TF: transfemoral.

In-hospital outcomes were available for all patients and are shown in Table 4 for the IPW-adjusted cohort, along with the results of the inverse probability of treatment weighted logistic regression. Primary and secondary end points for the overall population are shown in Supplementary Material, Table S2. In the IPW-adjusted cohort, the rate of any vascular complication was lower in the TC-TAVR group (3.5% vs 12%, OR: 0.26, 95%CI 0.07-0.95, *P* = 0.037), with a numerically reduction in the incidence of major vascular complications not reaching statistical significance (1.2% vs 4.5%, OR: 0.29, 95% CI: 0.06–1.38, *P* = 0.15). The lower rate of any vascular complication for the TCTAVR group remained after further adjustment by covariates with standardized mean differences > 0.1 despite IPW (OR: 0.27, 95%CI 0.07-0.99, P=0.05). There were no statistically significant differences between groups in the composite of life-threatening or major bleeding (3.8% TF-TAVR vs 2.8% TC-TAVR, *P* = 0.63), in-hospital mortality (1.5% TF- vs 2.8% TC-TAVR, *P* = 0.53) and stroke (0.4% TF- vs 1.2% TC-TAVR, *P* = 0.36; Graphic Abstract). The causes underlying vascular complications for both groups are detailed in Supplementary Material, Table S3. No cases of wound infection were observed among TC-TAVR recipients.

**Table 4:** In-hospital clinical outcomes for the propensity score-matched population

	IPW-adjusted population			
	TF (*n* = 442)	TC (*n* = 85)	*P*-Value	OR^a^ (95% CI)
Any vascular complication	53 (12)	3 (3.5)	0.037	0.26 (0.07–0.95)
Major vascular	20 (4.5)	1 (1.2)	0.15	0.29 (0.06–1.38)
Minor vascular	35 (7.9)	2 (2.4)	0.15	0.26 (0.04–1.64)
Life-threatening/major bleeding	17 (3.8)	2 (2.8)	0.63	0.70 (0.17–2.96)
Life-threatening bleeding	7 (1.6)	1 (1.2)	0.51	0.58 (0.12–2.88)
Major bleeding	11 (2.5)	2 (1.9)	0.73	0.73 (0.10–5.28)
All-cause mortality	6 (1.5)	2 (2.8)	0.53	1.78 (0.30–10.7)
Stroke	2 (0.4)	1 (1.2)	0.36	3.13 (0.43–23)
New permanent pacemaker	53 (12)	5 (5.3)	0.10	0.40 (0.13–1.19)
New-onset atrial fibrillation	31 (7)	3 (3.5)	0.21	0.47 (0.17–1.31)
Conversion to SAVR	0	0	NA	NA
Device success	414 (93.7)	80 (94.1)	0.98	0.98 (0.88–1.09)

Categorical data are expressed as *n* (%) with the *n* rounded up to the closes whole number.

a Inverse probability weighted logistic regression.

IPW: inverse probability of treatment weight; NA: not applicable; SAVR: surgical aortic valve replacement; TC: transcarotid; TF: transfemoral.

## DISCUSSION

To the best of our knowledge, this is the first report of a multicentre PS-matched comparison between TC and TF arterial accesses for TAVR in obese patients with a BMI of >35kg/m^2^. The main findings of our study may be summarized as follows: (i) in obese patients with severe aortic stenosis undergoing TAVR, the TC (versus TF) approach was associated with a lower incidence of vascular complications, and this finding was maintained after PS adjustment for baseline characteristics, valve type and sheath size, and (ii) TC-TAVR exhibited no significant differences compared to TF-TAVR regarding in-hospital mortality, major bleeding events and stroke, although there was a numerically higher rate of stroke in the TC group.

Stroke has been one of the most dreaded complications since the beginning of the TAVR era, and its incidence has remained rather constant over time [15]. Concern was raised about the feasibility and safety of TC-TAVR in relation to periprocedural cerebral ischaemic events. However, the stroke rate in TC-TAVR recipients has been reported to be <3% in previous series including both obese and normal-weight patients, with no significant differences when compared to the default TF access [12]. Our analysis showed that similar results were observed in an obese population, with stroke rates ranging between 2.4% and 1.2% for the overall and IPW-adjusted cohorts, respectively. Of note, the TC-TAVR group was highly selected from an anatomical standpoint, as normal flow and absence of significant carotid stenosis was demonstrated for every patient before TAVR by means of CT angiography and Doppler vascular sonography. Although there were not statistically significant differences between groups, our sample size was underpowered for detecting such differences in terms of relatively infrequent complications like stroke. In addition, a recent meta-analysis has suggested that, according to the pooled data, the TC approach may be linked to a higher rate of stroke events compared to the TF route [16]. Therefore, we should be cautious when interpreting our findings. Finally, it should be noted that the potential benefit of embolic protection devices was not assessed in our study.

**Figure 1:**
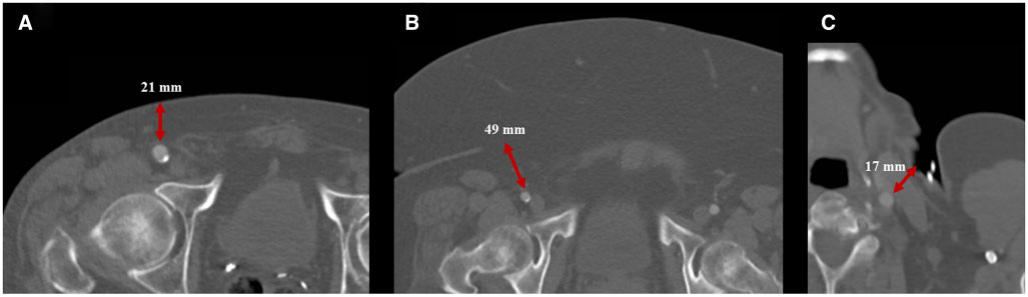
Features associated with transfemoral (**A** and **B**) and transcarotid (**C**) access. (**A**) Computed tomography image displaying an axial plane at the level of the right femoral artery in a normo-weight patient with a body mass index of 25.3 kg/m^2^. The distance between the skin and the anterior wall of the femoral artery was 21 mm (double-head red arrow) and no abdominal adipose panicle was observed. (**B**) Computed tomography image displaying an axial plane at the level of the right femoral artery in a morbid obese patient with a body mass index of 42.5 kg/m^2^. The distance between the skin and the anterior wall of the femoral artery was substantial (49 mm, double-head red arrow), and a huge abdominal adipose panicle was observed. (**C**) Computed tomography axial plane of the left common carotid artery, demonstrating its superficial location. BMI: body mass index; TC: transcarotid; TF: transfemoral.

The use of large bore catheters has been associated with high rates of vascular complications in TAVR recipients, and the reduction of the size of new-generation THV systems has translated into a significant decrease in such complications [17, 18]. In previous series of obese patients who underwent TAVR mainly by a TF route the rate of procedural-related vascular complications ranged between 10% and 16% [19–21], which was similar to the incidence observed in our study (12%). On the other hand, for patients with unsuitable TF access, the TC approach has been associated with a lower rate of major vascular complications when compared to transapical or transaortic access routes [10]. Our study is the first to suggest that, in obese patients without baseline unbalanced potential confounders, the TC access determined a lower risk of acute vascular complications. These results may be partly related to the intrinsic difficulty in managing and adequately positioning the percutaneous closure devices in patients exhibiting a large amount of fatty tissue (Fig. 1). In severely obese patients, the puncture site lies way deeper under the superficial skin than in patients with a normal phenotype, likely preventing a proper puncture of the anterior femoral artery wall in a high proportion of cases. In addition, the angulation of the gauge is not easy to determine in severely obese patients compared to non-obese patients. Finally, optimal manual compression of the femoral access proximal to the puncture site is more challenging and less frequently achieved in obese patients, hence impeding an adequate control of the access site during catheter and sheath exchanges. On the contrary, the TC access is more superficial and lies closer to the skin, ultimately facilitating the aforementioned manoeuvres (Fig. 1). It must be outlined that, even if the ProGlide system was the most frequently used for vascular closure in TF-TAVR patients, the use of the Prostar device was also important (>30%), and this device has been previously associated with poorer outcomes in terms of bleeding and vascular complications when compared to ProGlide [22].

There were no differences in major/life-threatening bleeding between TF and TC patients in our study, neither for the overall cohort nor for the PS-matched population. This fact implies that a substantial number of the observed vascular complications were either of ischaemic nature (e.g. artery dissection, acute thrombosis) or iatrogenic structural abnormalities involving the arterial wall (e.g. pseudoaneurysm) **(**Supplementary Material, Table S2**)**. The overall relatively low rate in major bleeding events in the TC subgroup (≈4%), which was similar than that observed in all-comers TC-TAVR series [12], highlights the feasibility and safety of this approach for severely obese patients. Early mortality rates were similar between groups, both overall and after adjustment. Nonetheless, given the low expected mortality in current TAVR series for patients with low and intermediate surgical risk, our study was statistically underpowered for this specific end point. Larger series are warranted to assess the role that the main vascular access plays in mortality among obese TAVR patients.

Finally, it should be outlined that other TAVR vascular approaches not evaluated in our investigation (i.e. subclavian access) may be perfectly suitable for obese patients. The subclavian access has demonstrated better outcomes than the more invasive transaortic and transapical routes [11, 12] and, when compared to the TC access, has yielded similar early rates for mortality, stroke and major complications in an overall TAVR population [23]. Besides, transsubclavian has been the most widely used alternative approach in the TVT registry for unsuitable TF cases [24]. In the concrete setting of obese TAVR receivers, there is a paucity of data and future studies are awaited. However, as for the TC route, transsubclavian TAVR may overcome most of the pitfalls associated with TF-TAVR in severe obese patients. Future studies are needed to assess long-term outcomes in the obese population.

### Limitations

This was an observational study with its inherent bias. TC-TAVR patients were derived exclusively from 3 tertiary centres with great experience with this approach and, consequently, these results may not be extrapolated to other centres or health systems. In addition, no information regarding alternative TAVR accesses beyond the TF approach was available for centres not performing TC-TAVR. The study was underpowered for certain end points (stroke and early mortality) derived from the available sample size, and some important clinical end points like anaesthesia-related complications and acute kidney injury were not assessed. Some variables potentially impacting outcomes (e.g. frailty status) were not recorded. However, the most important cardiovascular comorbidities and potential confounders were included in the PS analysis, and the quality of the matching enabled a well-balanced comparison.

## CONCLUSION

In patients with severe obesity undergoing TAVR with newer-generation devices, the TF approach was associated with a higher rate of vascular complications compared to the TC access, without statistically significant differences in early mortality and stroke rates between groups.

## SUPPLEMENTARY MATERIAL

Supplementary material is available at *ICVTS* online.

## Supplementary Material

ivab354_Supplementary_Data

## ABBREVIATIONS

BMI: Body mass index
IPW: Inverse probability of treatment weight
PS: Propensity score
TAVR: Transcatheter aortic valve replacement
TC: Transcarotid
TF: Transfemoral
THV: Transcatheter heart valve

## References

[1] Smith CR , Leon MB, Mack MJ, Miller DC, Moses JW, Svensson LG et al Transcatheter versus surgical aortic-valve replacement in high-risk patients. N Engl J Med 2011;364:2187–98. 21639811 10.1056/NEJMoa1103510

[2] Reardon MJ , Van Mieghem NM, Popma JJ, Kleiman NS, Søndergaard L, Mumtaz M et al; SURTAVI Investigators. Surgical or transcatheter aortic-valve replacement in intermediate-risk patients. N Engl J Med 2017;376:1321–31. 28304219 10.1056/NEJMoa1700456

[3] Mack MJ , Leon MB, Thourani VH, Makkar R, Kodali SK, Russo M et al Transcatheter aortic-valve replacement with a balloon-expandable valve in low-risk patients. N Engl J Med 2019;380:1695–705. 30883058 10.1056/NEJMoa1814052

[4] Hruby A , Hu FB. The epidemiology of obesity: a big picture. Pharmacoeconomics 2015;33:673–89. 25471927 10.1007/s40273-014-0243-x PMC4859313

[5] Wharton S , Lau DCW, Vallis M, Sharma AM, Biertho L, Campbell-Scherer D et al Obesity in adults: a clinical practice guideline. CMAJ 2020;192:E875–91. 32753461 10.1503/cmaj.191707 PMC7828878

[6] Sharma A , Lavie CJ, Elmariah S, Borer JS, Sharma SK, Vemulapalli S et al Relationship of body mass index with outcomes after transcatheter aortic valve replacement: results from the national cardiovascular data-STS/ACC TVT registry. Mayo Clin Proc 2020;95:57–68. 31902429 10.1016/j.mayocp.2019.09.027

[7] Ando T , Akintoye E, Trehan N, Telila T, Briasoulis A, Takagi H et al Comparison of in-hospital outcomes of transcatheter aortic valve implantation versus surgical aortic valve replacement in obese (body mass index ≥ 30 Kg/m2) patients. Am J Cardiol 2017;120:1858–62. 28860018 10.1016/j.amjcard.2017.07.098

[8] Cox N , Resnic FS, Popma JJ, Simon DI, Eisenhauer AC, Rogers C. Comparison of the risk of vascular complications associated with femoral and radial access coronary catheterization procedures in obese versus nonobese patients. Am J Cardiol 2004;94:1174–7. 15518615 10.1016/j.amjcard.2004.07.088

[9] Byrne J , Spence MS, Fretz E, Mildenberger R, Chase A, Berry B et al Body mass index, periprocedural bleeding, and outcome following percutaneous coronary intervention (from the British Columbia Cardiac Registry). Am J Cardiol 2009;103:507–11. 19195511 10.1016/j.amjcard.2008.10.027

[10] Chamandi C , Abi-Akar R, Rodés-Cabau J, Blanchard D, Dumont E, Spaulding C et al Transcarotid compared with other alternative access routes for transcatheter aortic valve replacement. Circ Cardiovasc Interv 2018;11:1–9. 10.1161/CIRCINTERVENTIONS.118.006388 30571205

[11] Junquera L , Kalavrouziotis D, Côté M, Dumont E, Paradis JM, DeLarochellière R et al Results of transcarotid compared with transfemoral transcatheter aortic valve replacement. J Thorac Cardiovasc Surg 2020;S0022-5223:30790–X. 10.1016/j.jtcvs.2020.03.091 32387164

[12] Watanabe M , Takahashi S, Yamaoka H, Sueda T, Piperata A, Zirphile X et al Comparison of transcarotid vs. transfemoral transcatheter aortic valve implantation. Circ J 2018;82:2518–22. 30068794 10.1253/circj.CJ-18-0530

[13] Campelo-Parada F , Rodés-Cabau J, Dumont E, Del Trigo M, Regueiro A, Doyle D et al A novel transcarotid approach for implantation of balloon-expandable or self-expandable transcatheter aortic valves. Can J Cardiol 2016;32:1575.e9-1575–1575.e12. 10.1016/j.cjca.2016.03.015 27181189

[14] Kappetein AP , Head SJ, Généreux P, Piazza N, van Mieghem NM, Blackstone EH et al Updated standardized endpoint definitions for transcatheter aortic valve implantation: the valve academic research consortium-2 consensus document. J Am Coll Cardiol 2012;60:1438–54. 23036636 10.1016/j.jacc.2012.09.001

[15] Auffret V , Lefevre T, Van Belle E, Eltchaninoff H, Iung B, Koning R et al Temporal trends in transcatheter aortic valve replacement in France: FRANCE 2 to FRANCE TAVI. J Am Coll Cardiol 2017;70:42–55. 28662806 10.1016/j.jacc.2017.04.053

[16] Faroux L. , L. Junquera, S. Mohammadi, D. Del Val, G. Muntané-Carol, et al Femoral Versus Nonfemoral Subclavian/Carotid Arterial Access Route for Transcatheter Aortic Valve Replacement: A Systematic Review and Meta-Analysis.J Am Heart Assoc 2020;9(19):e017460. 10.1161/JAHA.120.017460 PMC7792420 32990146

[17] Vendrik J , van Kesteren F, van Mourik MS, Piek JJ, Tijssen JG, Henriques JPS et al Procedural outcome and midterm survival of lower risk transfemoral transcatheter aortic valve implantation patients treated with the SAPIEN XT or SAPIEN 3 device. Am J Cardiol 2018;121:856–61. 29415808 10.1016/j.amjcard.2017.12.024

[18] Giannini C , De Carlo M, Tamburino C, Ettori F, Latib AM, Bedogni F et al Transcathether aortic valve implantation with the new repositionable self-expandable Evolut R versus CoreValve system: a case-matched comparison. Int J Cardiol 2017;243:126–31. 28595747 10.1016/j.ijcard.2017.05.095

[19] Abawi M , Rozemeijer R, Agostoni P, van Jaarsveld RC, van Dongen CS, Voskuil M et al Effect of body mass index on clinical outcome and all-cause mortality in patients undergoing transcatheter aortic valve implantation. Neth Heart J 2017;25:498–50. 28536936 10.1007/s12471-017-1003-2 PMC5571592

[20] Van Der Boon RMA , Chieffo A, Dumonteil N, Tchetche D, Van Mieghem NM, Buchanan GL et al Effect of body mass index on short- and long-term outcomes after transcatheter aortic valve implantation. Am J Cardiol 2013;111:231–6. 23102879 10.1016/j.amjcard.2012.09.022

[21] Koifman E , Kiramijyan S, Negi SI, Didier R, Escarcega RO, Minha S et al Body mass index association with survival in severe aortic stenosis patients undergoing transcatheter aortic valve replacement. Cathet Cardiovasc Intervent 2016;88:118–24. 10.1002/ccd.26377 26715505

[22] Berti S , Bedogni F, Giordano A, Petronio AS, Iadanza A, Bartorelli AL, Italian Society of Interventional Cardiology‐GISE† et al Efficacy and safety of ProGlide versus Prostar XL vascular closure devices in transcatheter aortic valve replacement: the RISPEVA registry. J Am Heart Assoc 2020;9:e018042. 33103545 10.1161/JAHA.120.018042 PMC7763424

[23] Amer MR , Mosleh W, Joshi S, Mather JF, El-Mallah W, Cheema M et al Comparative outcomes of transcarotid and transsubclavian transcatheter aortic valve replacement. Ann Thorac Surg 2020;109:49–56. 31279787 10.1016/j.athoracsur.2019.05.035

[24] Dahle TG , Kaneko T, McCabe JM. Outcomes following subclavian and axillary artery access for transcatheter aortic valve replacement: Society of the Thoracic Surgeons/American College of Cardiology TVT Registry Report. JACC Cardiovasc Interv 2019;12:662–9. 30947940 10.1016/j.jcin.2019.01.219

