# Supplementary material for: Transcatheter aortic valve replacement in obese patients: procedural vascular complications with the trans-femoral and trans-carotid access routes
Source: Interact Cardiovasc Thorac Surg. 2021 Dec 23;34(6):982–9. doi: 10.1093/icvts/ivab354 (PMC9159419; doi:10.1093/icvts/ivab354)
Supplement: ivab354_Supplementary_Data [file ivab354_supplementary_data.docx]

**Supplemental Table 1:** List of inclusion and exclusion criteria.

| **INCLUSION CRITERIA** |
| --- |
| Body mass index > 35 kg/m2 |
| Underwent transcarotid or transfemoral TAVR |
|  |
| **EXCLUSION CRITERIA** |
| Lack of clinical information on early outcomes |
| Use of early-generation transcatheter heart valves: i.e., SAPIEN, SAPIEN XT and CoreValve |
| For Trans-Femoral cases:  - Surgical cut-down for transfemoral approach |
| For Trans-Carotid cases:  - Diseased common carotid artery for main TAVR access.  - Presence of contralateral significant (>50%) common or internal carotid artery stenosis |

**Supplemental Table 2: Number of TC-TAVR patients included from each of the centers.**

| **Center** | **N** |
| --- | --- |
| Institute Universitaire de Cardiologie et Pneumologie de Quebec | 51 |
| Centre Hospitalier Universitaire de Lille | 26 |
| Hôpital européen Georges-Pompidou, Paris | 8 |

**Supplemental Table 2**: Primary and secondary outcomes in the overall population.

|  | Overall population | | |
| --- | --- | --- | --- |
|  | TF  (n=454) | TC  (n=85) | p  value |
| Any vascular complication  - Major vascular  - Minor vascular | 56 (12.3)  21 (4.6)  37 (8.2) | 4 (4.7)  2 (2.4)  2 (2.4) | 0.04  0.34  0.06 |
| Life-threatening/Major bleeding  - Life threatening bleeding  - Major bleeding | 18 (4)  7 (1.5)  12 (2.6) | 3 (3.5)  2 (2.3)  1 (1.2) | 0.85  0.64  0.70 |
| All-cause mortality | 8 (1.8) | 2 (2.4) | 0.71 |
| Stroke | 3 (0.7) | 2 (2.4) | 0.17 |
| New permanent pacemaker | 54 (11.9) | 5 (5.9) | 0.12 |
| New-onset atrial fibrillation | 33 (7.8) | 6 (7.1) | 0.85 |
| Conversion to SAVR | 0 | 0 | NA |

SAVR: surgical aortic valve replacement. TC: transcarotid. TF: transfemoral

**Supplemental Table 3**: Causes of vascular complication for the TF- and TC-TAVR groups.

|  | TF-TAVR  (n=56) |  | TC-TAVR  (n=4) |
| --- | --- | --- | --- |
| Ileo-femoral dissection | 14 (25) | Carotid dissection repaired during procedure | 1 (25) |
| Femoral hematoma | 14 (25) | Carotid dissection requiring reintervention | 1 (25) |
| Femoral perforation | 7 (12.5) | Cervical hematoma | 2 (50) |
| Femoral stenosis | 6 (10.7) |  |  |
| Closure device failure requiring stent implant | 5 (8.9) |  |  |
| Femoral Pseudoaneurysm | 5 (8.9) |  |  |
| Aortic rupture/dissection | 4 (7.1) |  |  |
| Femoral thrombosis | 1 (1.8) |  |  |

TAVR: transcatheter aortic valve replacement. TC: transcarotid. TF: transfemoral.

**Supplemental Figure 1**. Box plots of the propensity score values before and after propensity-matching for TF-TAVR and TC-TAVR groups.


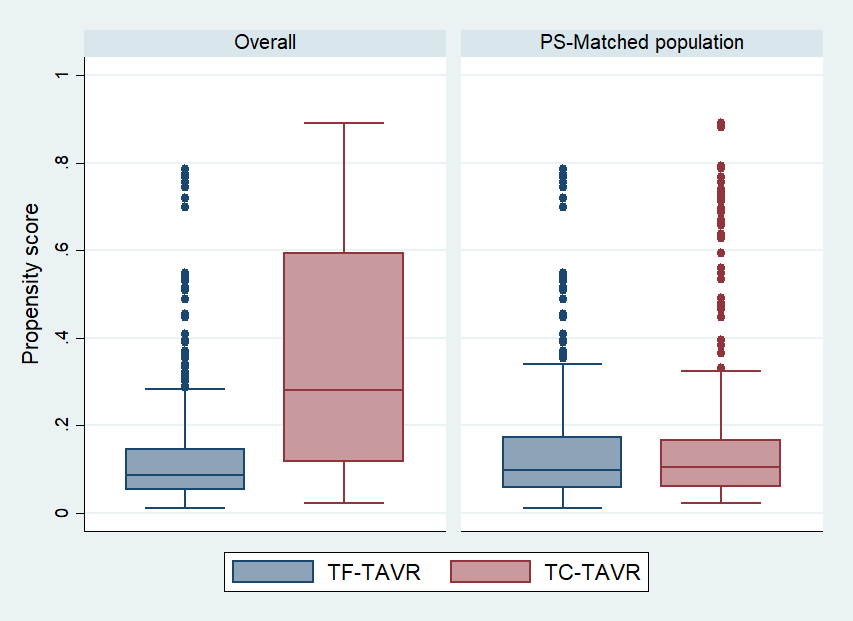


PS: propensity-score. TAVR: transcatheter aortic valve replacement. TC: transcarotid. TF: transfemoral.

**Supplemental Figure 2**. Cumulative graphic representation of the propensity score values before and after inverse probability of treatment weighted-matching for the TF-TAVR and TC-TAVR groups.


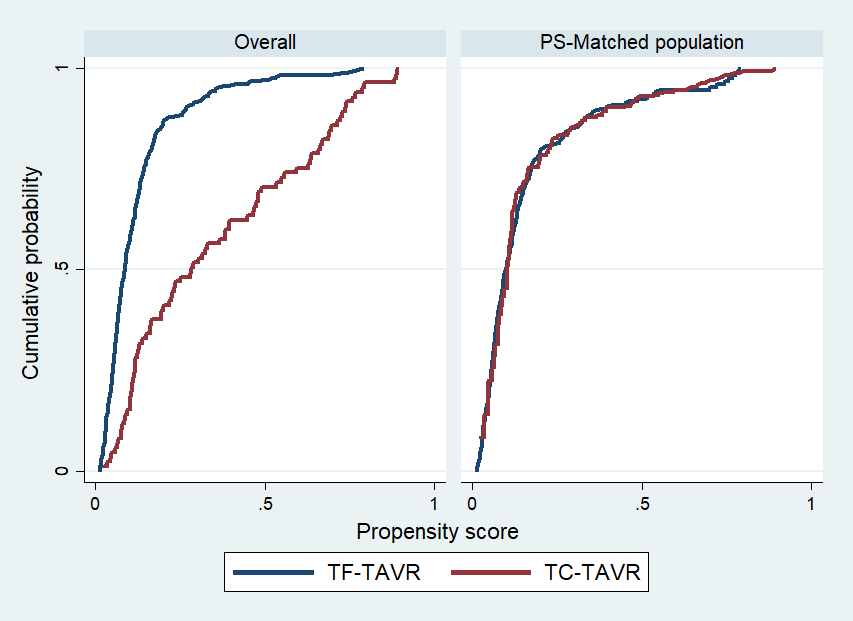


PS: propensity-score. TAVR: transcatheter aortic valve replacement. TC: transcarotid. TF: transfemoral.
